# Supplementary figures and images for: Sensors for Expert Grip Force Profiling: Towards Benchmarking Manual Control of a Robotic Device for Surgical Tool Movements
Source: Sensors (Basel). 2019 Oct 21;19(20):4575. doi: 10.3390/s19204575 (PMC6848933; doi:10.3390/s19204575)

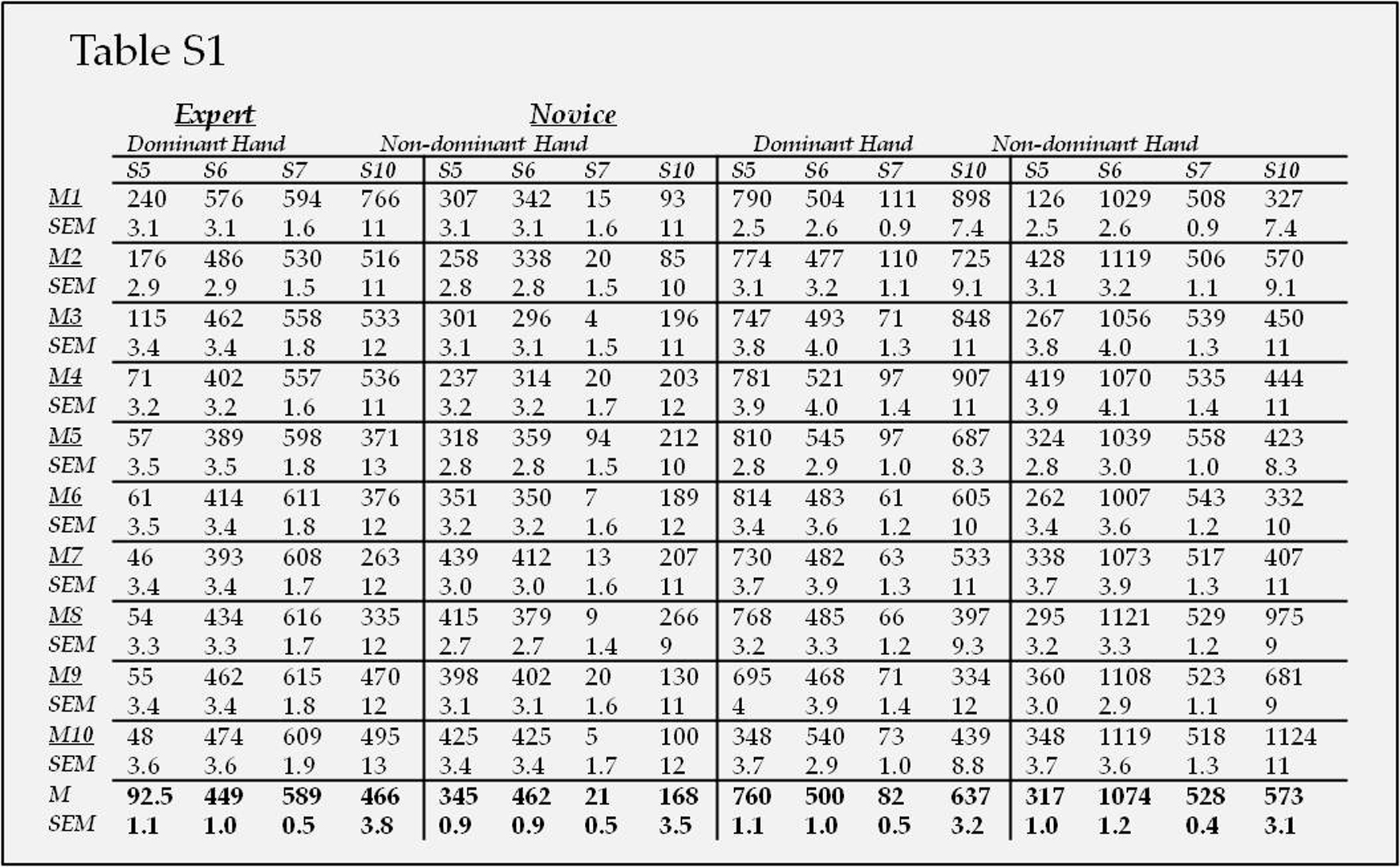

Supplement: Supplementary file 1 [file sensors-19-04575-s001.zip › sensors-569027-sl-new/SupplementaryS1-S2/TableS1.jpg]
